# Picking Up on Preservatives: New Biomarkers for Gauging Paraben Exposure

**Published:** 2006-12

**Authors:** Ernie Hood

As scientists seek to characterize human exposures to chemicals, they need more valid, accurate biomarkers—telltale molecular signatures that indicate a particular exposure has occurred. A team from the CDC has now provided the field with a new biomarker that could help researchers document exposures to a class of antimicrobial preservatives called parabens **[*EHP* 114:1843–1846; Ye et al.]**.

Parabens are used in shampoos, cosmetics, moisturizers, medications, foods, and beverages. Concerns have arisen about the potential human health risks associated with the widespread use of these weakly estrogenic compounds, including widely publicized speculation that parabens in antiperspirants were linked to breast cancer. Although that theory was later refuted, frequent and common human exposure to parabens means that research attention will continue to focus on these chemicals.

Until now, the only biomarker used for human paraben exposure was *p*-hydroxybenzoic acid in urine. But that metabolite is produced by the hydrolysis of all of the various paraben compounds, so it is nonspecific to individual parabens, which vary widely in estrogenic bioactivity.

The CDC team measured the presence of free and conjugated parent parabens in urine to determine their suitability to be biomarkers of human exposures. They analyzed the urinary concentrations of methyl, ethyl, *n*-propyl, butyl (*n*- and *iso*-), and benzyl parabens in 100 human adults with no known industrial exposure to the compounds. The results appear to support the viability of those measures as biomarkers of exposure.

Methyl and *n*-propyl parabens, the parabens most commonly used in cosmetics and foods, were found at the highest median concentrations in almost all the samples—99% contained the former and 96% the latter. The authors say this could result from the widespread use of these compounds; from differences in the absorption, distribution, metabolism, and excretion of the various parabens; or from a combination of both factors. Other parent compounds, such as ethyl and butyl paraben, appeared in more than half of the samples.

Regardless of the reason for such high frequencies of detection, the researchers say their results suggest that urinary parabens and their conjugates could be valid biomarkers of exposure to these chemicals. The detection and measurement methodologies used by the CDC scientists could help investigators as they seek to characterize the potential health risks associated with exposure to the individual paraben compounds.

## Figures and Tables

**Figure:**
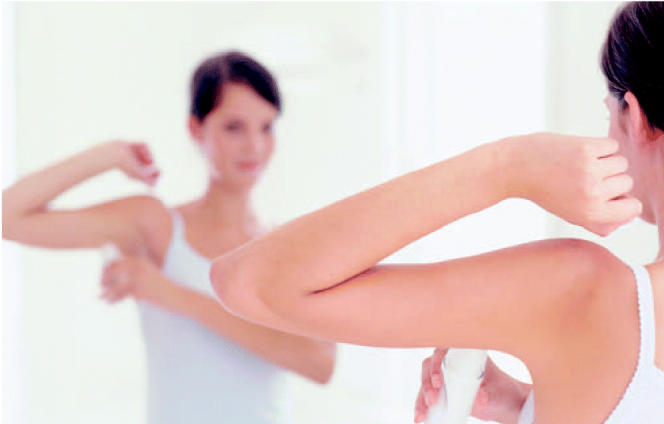
Biomarkers roll on Scientists may have discovered a new way to measure exposure to parabens, an ingredient in many toiletries and cosmetics.

